# RPSLearner: A Novel Approach Based on Random Projection and Deep Stacking Learning for Categorizing Non-Small Cell Lung Cancer

**DOI:** 10.1002/aisy.202500635

**Published:** 2025-10-05

**Authors:** Xinchao Wu, Jieqiong Wang, Shibiao Wan

**Affiliations:** 1. Department of Genetics, Cell Biology and Anatomy, University of Nebraska Medical Center, Omaha, NE 68198, USA; 2. Department of Neurological Sciences, University of Nebraska Medical Center, Omaha, NE 68198, USA

**Keywords:** lung cancer subtype prediction, machine learning, random projection, stacking learning, transcriptomics

## Abstract

Non-small cell lung cancer (NSCLC) comprises the largest subtype of lung cancer with the most cases. Lung adenocarcinoma and lung squamous cell carcinoma are two NSCLC subtypes that pose challenges for accurate diagnosis using conventional methods, including histological examination and imaging, which can be slow and inconclusive. To address these concerns, RPSLearner is proposed, which combines random projection (RP) for dimensionality reduction and stacking ensemble learning to accurately predict lung cancer subtypes. Specifically, multiple independent RP matrices are first generated to project the high-dimensional RNA-seq data into a lower-dimensional space, whose features are subsequently concatenated. After that, the concatenated RP features are fed into a stack of diverse base classifiers, and integrated the predictions from base models via a deep linear layer network. Benchmarking tests on 1 333 NSCLC patients demonstrated that RPSLearner outperformed state-of-the-art approaches for lung cancer subtype classification. Specifically, RPSLearner efficiently preserved sample-to-sample distances even after significant dimension reduction, and the meta-model in RPSLearner yielded consistently higher scores than individual base models. In addition, the feature fusion method outperformed conventional score ensemble methods. We believe RPSLearner is a promising model for downstream lung cancer clinical diagnosis, and it holds the potential to be extended to subtyping of other types of cancer.

## 1. Introduction

Lung cancer remains one of the leading causes of cancer-related mortality worldwide, accounting for a substantial proportion of cancer diagnoses and deaths each year.^[1,2]^ Nonsmall cell lung cancer (NSCLC) constitutes ≈85% of all lung cancer cases, with subtypes such as lung adenocarcinoma (LUAD) and lung squamous cell carcinoma (LUSC), exhibiting distinct molecular and clinical characteristics.^[3]^ Accurate identification of NSCLC subtypes is critical for prognosis determination, therapeutic decision-making, and the development of personalized treatment strategies.^[4]^ Traditional diagnostic methods, including histopathological examination and imaging techniques,^[5]^ while valuable, often lack the precision required for nuanced subtype differentiation, particularly in early-stage disease.^[6]^ Besides, histological examination increases the risk of lung cancer metastasis due to physical damage.^[7]^

In recent years, advancements in high-throughput genomic and transcriptomic technologies have revolutionized our understanding of the molecular mechanisms of lung cancer, enabling the identification of specific biomarkers and genetic signatures associated with different NSCLC subtypes.^[8]^ Among these techniques, transcriptomic profiling provides a dynamic and quantitative snapshot of gene expression, thereby serving as a more direct indicator of the cellular functional state.^[9]^ However, the analysis of such high-dimensional omics data presents significant computational and methodological challenges. The complexity and volume of transcriptomic profiles require robust feature selection and dimensionality reduction techniques to extract meaningful patterns while mitigating the risk of overfitting and enhancing model generalizability.^[10]^

Machine learning (ML) approaches have emerged as powerful tools for integrating and analyzing large-scale biological data, offering the potential to improve the accuracy and efficiency of lung cancer subtype prediction. For instance, Huang et al.^[11]^ utilized logistic regression (LR) with LASSO shrinkage for predicting NSCLC subtypes based on RNA-seq data. Their model demonstrated superior performance compared to LR using principal components and K-nearest neighbor (KNN) algorithms. Similarly, Yuan et al.^[12]^ applied a method called Monte Carlo feature selection (MCFS) in combination with a support vector machine (SVM) for subtype stratification based on micro-array gene expression data, showing better results than the random forest (RF) classifier. Although these studies illustrated the effectiveness of ML in NSCLC subtype classification, significant challenges remain. In particular, the high dimensionality of omics data can lead to overfitting and reduced generalizability, and intersample variability further complicates the creation of robust predictive models.^[13]^ Recently, we have witnessed the significant development of multiple deep learning approaches^[14,15]^ tailored to cancer subtype diagnosis, combining histological images and genomics data, which have demonstrated promising results for cancer subtyping. However, these types of methods require the availability of transcriptomics data paired with histological staining imaging data, which are very costly or laborious to obtain. In this case, we focus on developing a novel approach solely based on transcriptomics data for cost-effective and accurate NSCLC subtype classification.

To address these challenges, we developed RPSLearner, combining random projection (RP) for dimensionality reduction and stacking ensemble learning to accurately predict lung cancer subtypes. In detail, by leveraging multiple independent RP matrices and fusing these reduced features, RPSLearner can capture essential sample-to-sample distances.^[16,17]^ Subsequently, RPSLearner uses a stacking learning framework to integrate diverse heterogeneous base classifiers with a deep linear network as a *meta*-model.^[18,19]^ The stacking framework not only consolidates complementary predictive insights but also yields improved performance metrics over conventional methods. Ultimately, in this study, we demonstrated that RPSLearner significantly enhances NSCLC subtype classification and offered a versatile framework that may be adapted for other cancer subtyping applications.

## 2. Results

### 2.1. The Design of RPSLearner

**Figure 1.**
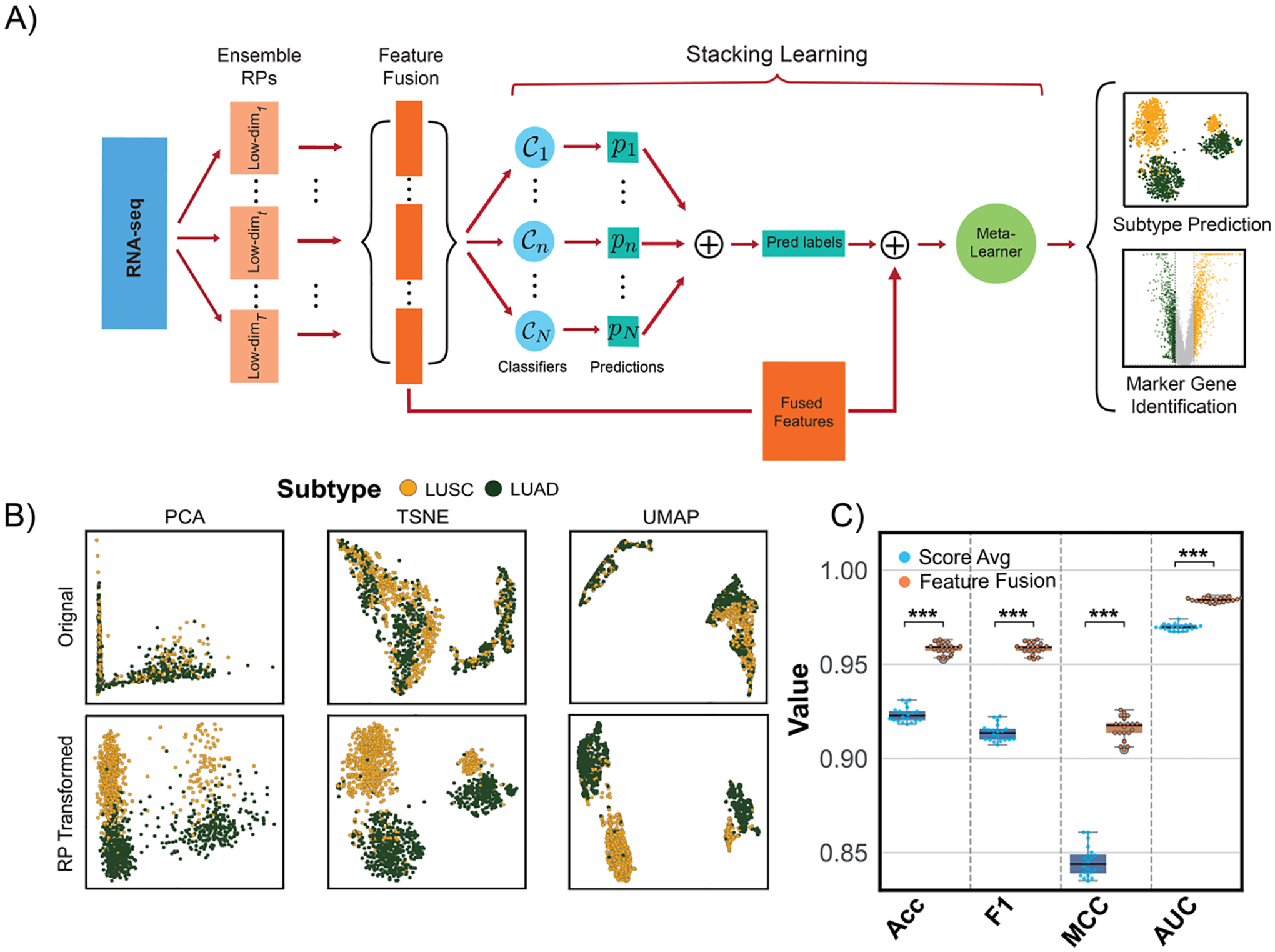
The overview of lung cancer TCGA datasets and the RPSLearner algorithm. A) The computational pipeline of the RPSLearner. Log-transformed RNA-seq data multiplied by a series of independent RP matrices, resulting in multiple low-dimensional representations of the input data, following concatenation of these representations named as feature fusion. After that, within the stacking learning framework, concatenated features were fed into diverse base classifiers, generating independent prediction labels. The input of the *meta*-learner is the concatenation of concatenated features with the prediction labels of base classifiers, and the final predicted results were used for subtype prediction and further marker gene identification. B) Low-dimension visualization of original data and RP-transformed data through diverse techniques. C) The performance of the score average strategy against feature fusion. Statistical significance level denoted as: ns (not significant) or *P* ≥ 0.05, * for *P* < 0.05, ** for *P* < 0.01, *** for *P* < 0.001. RP: RP. Low-dim: low-dimension representations. Pred labels: prediction labels. PCA: Principal Component Analysis. t-SNE: t-distribution stochastic neighboring embedding. UMAP: Uniform manifold approximation projection. Score Avg: score averaging. Acc: accuracy. MCC: Matthews Correlation Coefficient. AUC: Area under the ROC curve.

To accurately identify subtypes of NSCLC, we presented RPSLearner, a novel computational framework that integrates RP with stacking ensemble learning. The whole process of the method was shown in Figure 1A. RPSLearner leverages RP’s capabilities of dimensionality reduction to deal with the high-dimensional nature of transcriptomic data.^[20]^ Specifically, it applied multiple RPs to patients’ transcriptomic profiles to extract meaningful hidden information, significantly decreasing the feature space while preserving the intrinsic distances between samples. This approach facilitated the handling of large-scale omics datasets, ensuring computational efficiency without compromising the integrity of the underlying biological signals.

**Figure 2.**
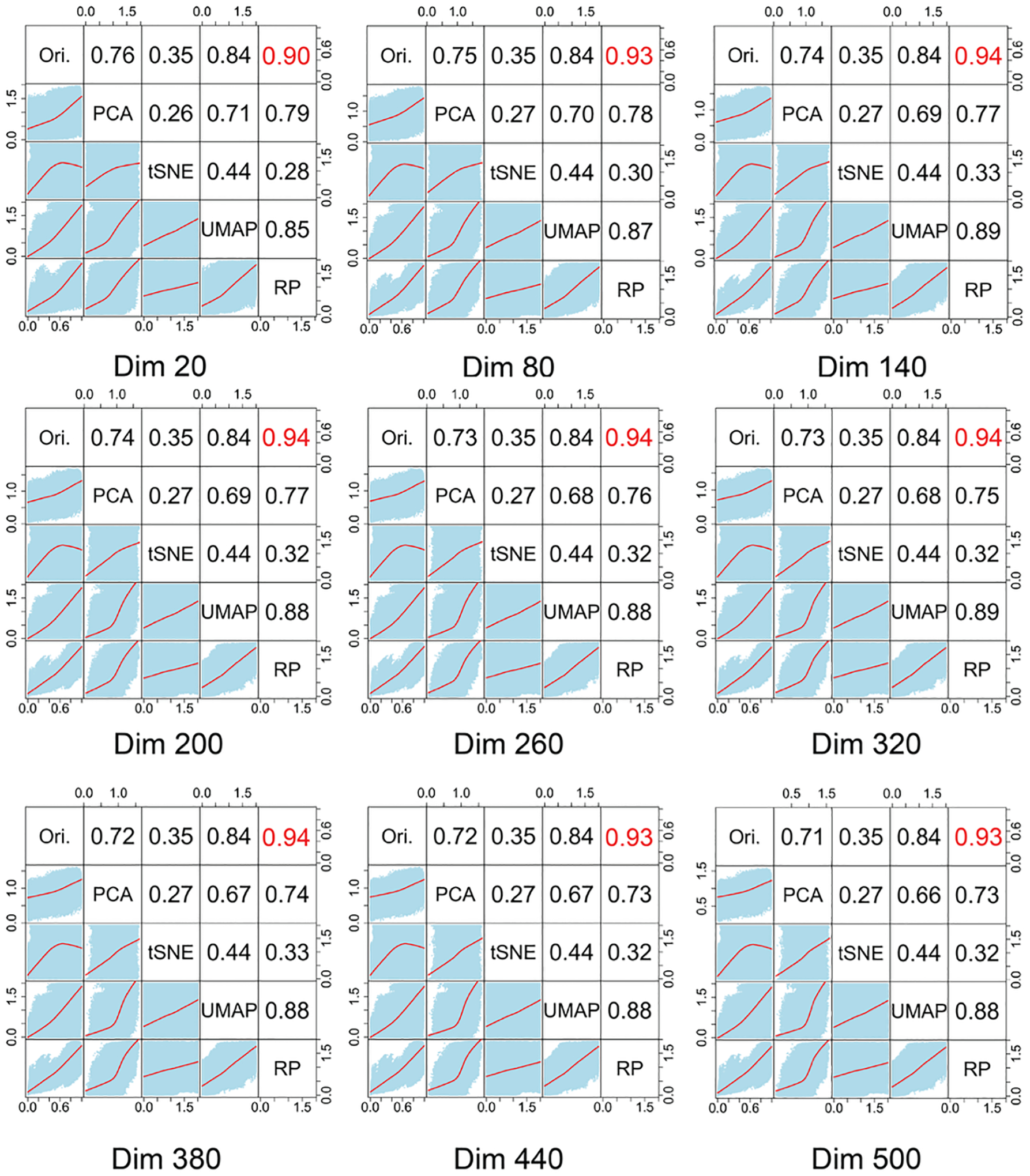
Correlation analysis across diverse dimensionality reduction methods. These figures demonstrate the performance of RP with other commonly used dimension-reduction techniques across different dimensions in ascending order. The upper triangular plots exhibited the Pearson correlation coefficients between different feature vectors, where the upper right indicates the coefficient of the original vector and RP-transformed vector, presenting the highest among all pairs. The lower triangular plots illustrated the fitting curve over the sample-to-sample distance across different feature vector pairs, highlighting the robustness and effectiveness of the RP method. Ori.: Original feature vector. PCA: Principal Component Analysis. t-SNE: t-distribution stochastic neighboring embedding. UMAP: Uniform manifold approximation projection. RP: RP.

Unlike standard “reduce-then-classify” pipelines, RPSLearner is distinguished by methodological innovations as: 1) it ensembles multiple independent RP matrices to create diversified, low-correlated views of the data for comprehensive information extraction; 2) it combines heterogeneous base learners under a *meta*-learner architecture, which pools complementary decision boundaries, leveraging capabilities of different model families; and 3) it retains pairwise distance more faithfully than conventional dimension-reduction methods like PCA, t-SNE, or UMAP, as shown in our correlation preservation analysis (Figure 2). Practically, RP avoids reoptimizing nonlinear objectives and yields strong accuracy-to-cost trade-offs on small-*n*, large-*p* data conditions under which deep-learning-based models will easily overfit.

### 2.2. RPSLearner Efficiently Preserved Sample-to-Sample Distances

To demonstrate the effectiveness of RP, we applied PCA,^[21]^ t-SNE,^[22]^ and UMAP^[23]^ to visualize the RP-transformed NSCLC RNA-seq data and compared them against the original data (Figure 1B). Across diverse techniques, the RP-transformation, fusing multiple RP dimension reduction results, exhibited clear subtype identities with distinct cluster groups, suggesting that effectively extract meaningful features in lung cancer subtype prediction and simplify the prediction task.

To demonstrate the effectiveness of the stacking learning method in RPSLearner, we compared two ways of leveraging multiple RPs, feature fusion and score ensemble. Conventionally, methods applied to score ensembles, which average the prediction probabilities from independent runs as the final score. Feature fusion concatenates RP-transformed features for each randomly generated feature and feeds them into the stacking learning pipeline. We compared the performance between modified RanBALL,^[16]^ which was originally used for leukemia subtypes prediction, applied an ensemble strategy, and RPSLearner, using feature fusion. Through 20 times fivefold cross-validation (CV), we demonstrated feature fusion yields statistically significantly higher accuracy (*p*-value = 6.74e–6), F1 score (*p*-value = 6.32e–8), Matthews Correlation Coefficient (MCC, *p*-value = 1.05e–7), and area under the ROC curve (AUC, *p*-value = 2.47e–4) than score ensemble (Figure 1C). To demonstrate the robustness of the superiority of our feature-fusion model against conventional score ensemble models, we used a 20% hold-out set for testing and the remaining 80% for training to compare these two models. Results (Figure S2, Supporting Information) suggested that the feature-fusion model consistently outperformed the score-ensemble model across all dimensions from 100, 200, 400, 600, to 800. The improvement of the feature-fusion model over the score-ensemble model was more significant at lower RP dimensions. These results suggested that integrating projected features directly exploits complementary representations more efficiently than simple probability averaging.

To further validate the performance of RP for dimensionality reduction, we next evaluated RP against PCA, t-SNE, and UMAP for NSCLC RNA-seq data. Figure 2b compares Pearson correlation coefficients and the distance-fitting curve between the original and projected feature vectors. RP consistently maintained higher correlations across various dimensions up to 0.94, more effectively preserving sample-to-sample relationships. UMAP showed the second-best correlation (0.84) but lagged RP. PCA and t-SNE were less effective in preserving pairwise distances for this dataset. These findings underscore RP’s suitability for retaining meaningful data patterns in a reduced-dimension space.

### 2.3. Stacking Meta Learning Outperforms Individual Base Learning Models

To find the optimal configurations of the dimensionality and the number of RP applications, we performed a grid search to find the optimized hyperparameter. We first tested RPSLearner using different projected dimensions (from 100 to 1,400) to find optimal performance (Figure S3A, Supporting Information). With 10 times fivefold CV, it demonstrated that the accuracy and other metrics improved from 100 to 400 dimensions and then stabilized. Subsequently, we explored using multiple independent RP matrices (Figure S3B, Supporting Information). With the same evaluation setting, it showed that accuracy and other metrics steadily rose as the number of RPs used increased up to around 25, reaching 0.961 in accuracy, 0.96 in F1, 0.92 in MCC, and 0.986 in AUC on average. Beyond 25 RPs, the performance gains were marginal, suggesting that moderate RP ensembles sufficiently capture complex signals. Performance plateaued around 400 dimensions and 10–20 RP matrices. Therefore, we chose to use 400 dimensions and 20 applications of RPs.

**Figure 3.**
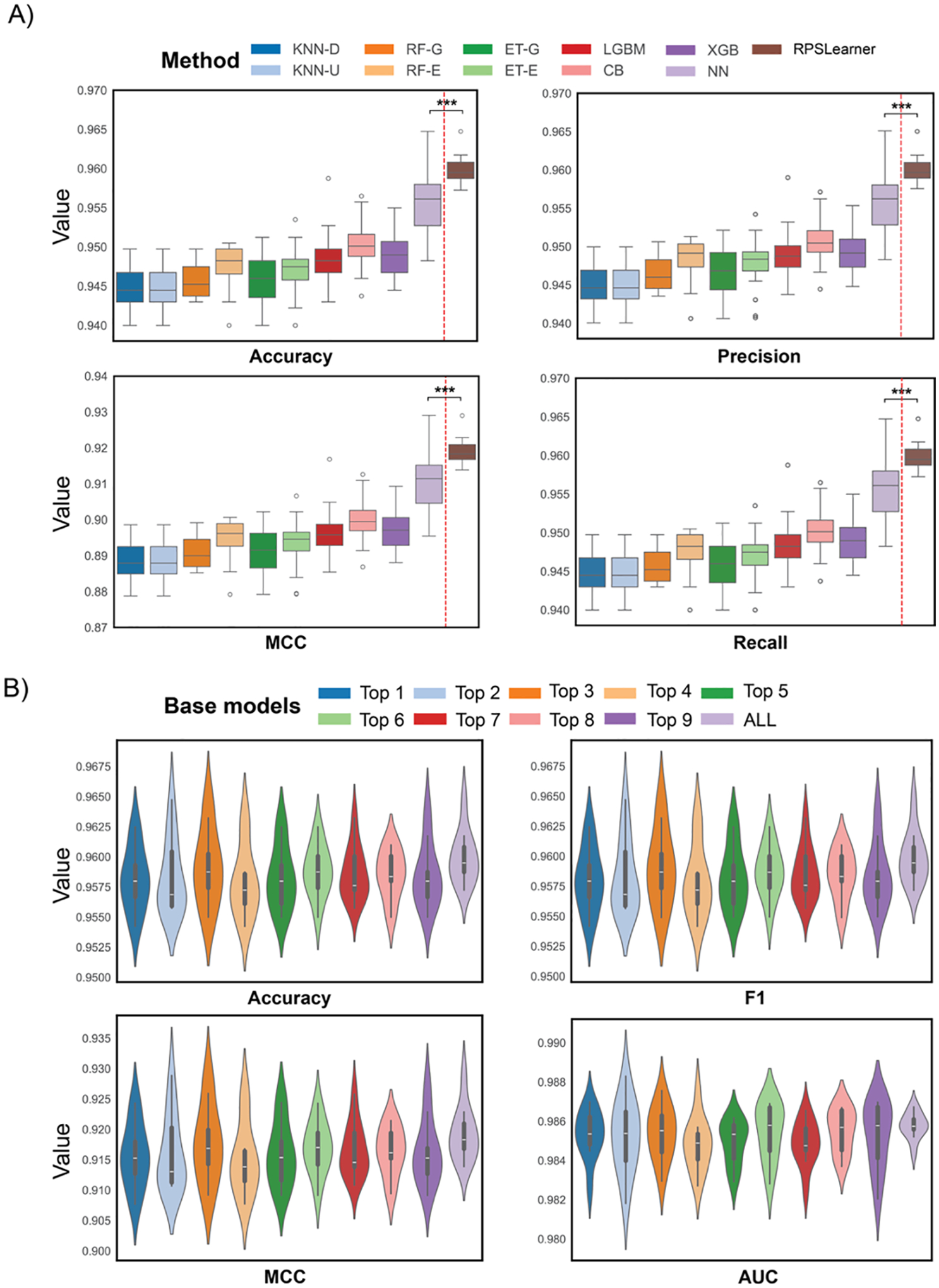
The performance of RPSLearner in lung cancer subtype prediction. A) The comparison of the performance of diverse individual base models and stacked models, RPSLearner. B) The comparison of the performance of various combinations of base models in stacking learning, top k selection was based on the results of previous individual base model comparison, ALL represented using all ten models. KNN-D: KNN in distance metrics, KNN-U: KNN in uniform metrics, RF-G: RF in Gini metrics, RF-E: RF in entropy metrics, ET-G: ExtraTree in Gini metrics, ET-E: ExtraTree in entropy metrics, LGBM: LightGBM, CB: CatBoost, XGB: XGBoost, NN: neural network. Statistical significance level denoted as: ns (not significant) or *P* ≥ 0.05, * for *P* < 0.05, ** for *P* < 0.01, *** for *P* < 0.001.

To evaluate the performance of stacking learning, we compared the performance of each individual base model and the NN-based *meta*-model. We assessed them using 10 times fivefold CV with 20 individual 400-dimensional RP matrices. Figure 3A showed *meta*-model achieved the best performance across all metrics. The NN base classifier performed the second best, but stacking a *meta*-model delivered a consistent and significant boost in accuracy (*p*-value = 5.79e–4), F1 (*p*-value = 4.55e–4), MCC (*p*-value = 4.55e–4), and AUC (*p*-value = 5.79e–4).

To deeper examine the effectiveness of stacking learning, we compared the performance across various composite of base models used. We assessed with the same RP setting as above, and Figure 3B exhibited that the performance of using all base models obtained the smallest variance, which was no less than the best performance of base model combination using the top 3. In other words, it enhanced the robustness of the model prediction.

### 2.4. RPSLearner Outperformed State-of-the-Art Approaches for Lung Cancer Subtype Identification

**Figure 4.**
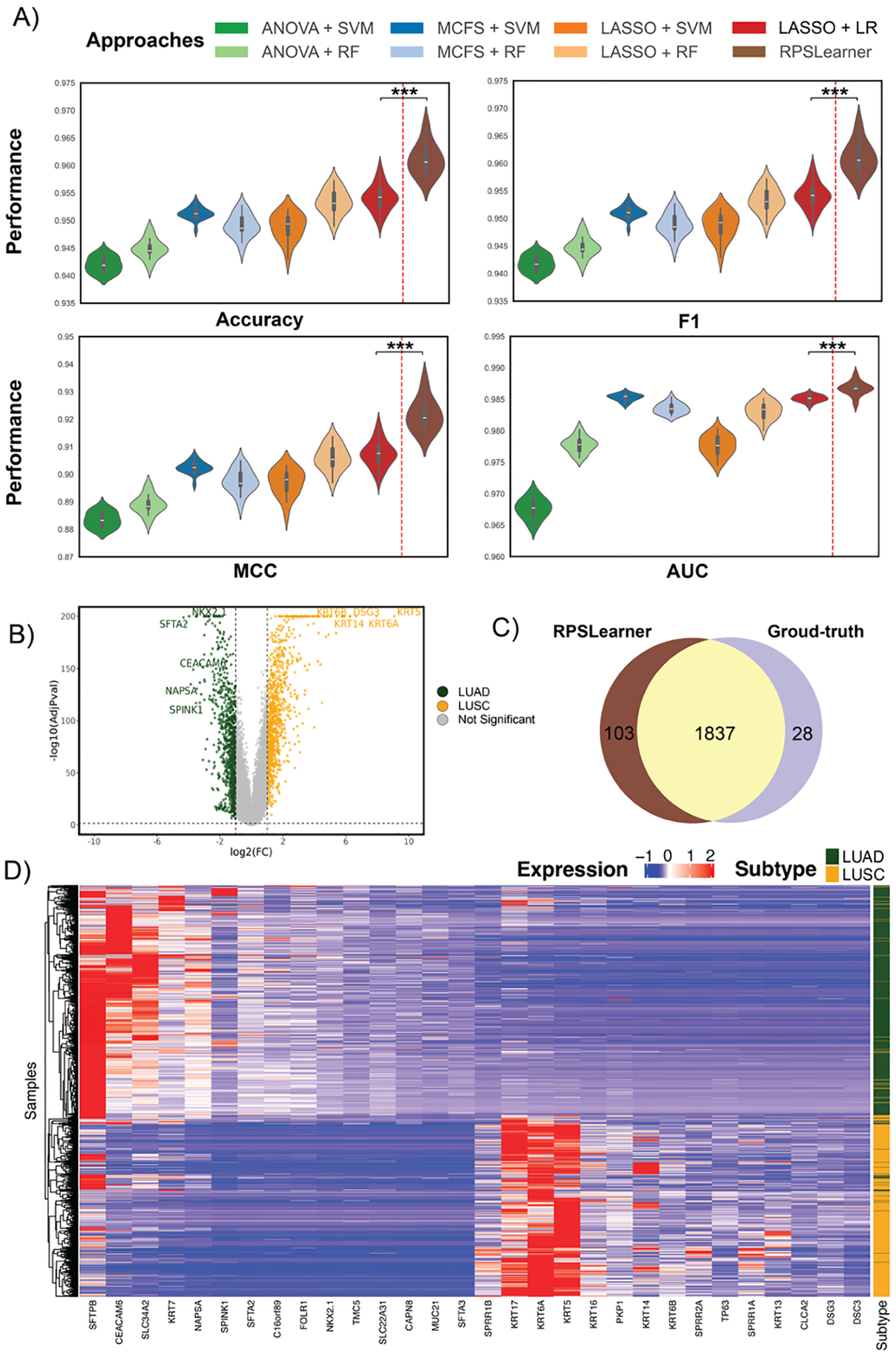
RPSLearner outperforms state-of-the-art methods with interpretable results. A) The benchmark of methods for lung cancer subtype prediction. B) The volcano plot illustrates the significance of the DE genes of each subtype. C) Comparison among DEGs identified based on RPSLearner and ground-truth, revealing the method-specific DEGs and their overlap. D) Expression of the DEGs extracted from RPSLearner prediction. Statistical significance level denoted as: ns (not significant) or *P* ≥ 0.05, * for *P* < 0.05, ** for *P* < 0.01, *** for *P* < 0.001. ANOVA: analysis of variance. SVM: support vector machine. RF: RF. MCFS: MCFS. LASSO: least absolute shrinkage and selection operator. LR: linear regression.

To demonstrate that RPSLearner could outperform state-of-the-art methods, we benchmarked RPSLearner against previously published methods for NSCLC subtype prediction (Figure 4A). Approaches include MCFS + SVM, MCFS + RF, Lasso + SVM, Lasso + RF, ANOVA + SVM,^[24]^ ANOVA + RF, and a Lasso-based linear regression model developed by Huang et al.^[11]^ obtained accuracy ranging from around 0.93 to 0.95. By contrast, RPSLearner achieved around 0.96 accuracy, which is significantly higher (*p*-value = 1.93e–6) than the SOTA method. The consistent and significant improvement was also shown in other metrics (F1 *p*-value = 3.27e–6, MCC *p*-value = 1.25e–6, AUC *p*-value = 6.98e–5). We further compared RSPLearner with one of the state-of-the-art deep learning architectures, i.e., autoencoder (AE). Specifically, we designed the AE as a 2-layer encoder and 2-layer decoder (latent dim 256), followed by a 2-layer multilayer perceptron (MLP) classifier trained end-to-end. Training details mirror the neural network *meta*-learner settings (optimizer: Adam, learning rate: 1e–4, epochs: 1000). Across all four metrics (i.e., Accuracy, F1-score, MCC, and AUC), RPSLearner consistently outperformed the AE method on five times fivefold CV tests. This is because cancer (such as NSCLC) patient data are usually with a small sample size but with very high-dimensional features, for which the deep learning architecture like AE might not be suitable to tackle. On the contrary, our approach RPSLearner, leverages a joint strategy of RP and stacking models, which could efficiently extract more meaningful biological signals and boost the performance by incorporating robust but diverse information derived from multiple RPs.

Besides, we further identify differential expression genes (DEGs) based on RPSLearner prediction (Figure 4B,D). There are many well-established biomarkers of LUAD and LUSC included in the identified DEGs. For example, KRT5, KRT6A, KRT14, and DSG3 (All of these genes shown are with an adjusted *p*-value < 1e–200) in LUSC,^[25]^ and NKX2.1,^[26]^ SFTA2^[27]^ (All of these genes shown are with an adjusted *P*-value < 1e–200) in LUAD have been evidently proved directly correlated to the lung cancer diagnosis and progression. Additionally, we compared the DEGs identified with the prediction of other methods and ground-truth (Figure 4C). Through RPSLearner prediction, there is a larger overlap between RPSLearner with ground-truth, and RPSLearner could find more DEGs than ground-truth. These findings highlight the accuracy of the prediction, as well as potential novel molecular signatures identified through RPSLearner prediction.

**Figure 5.**
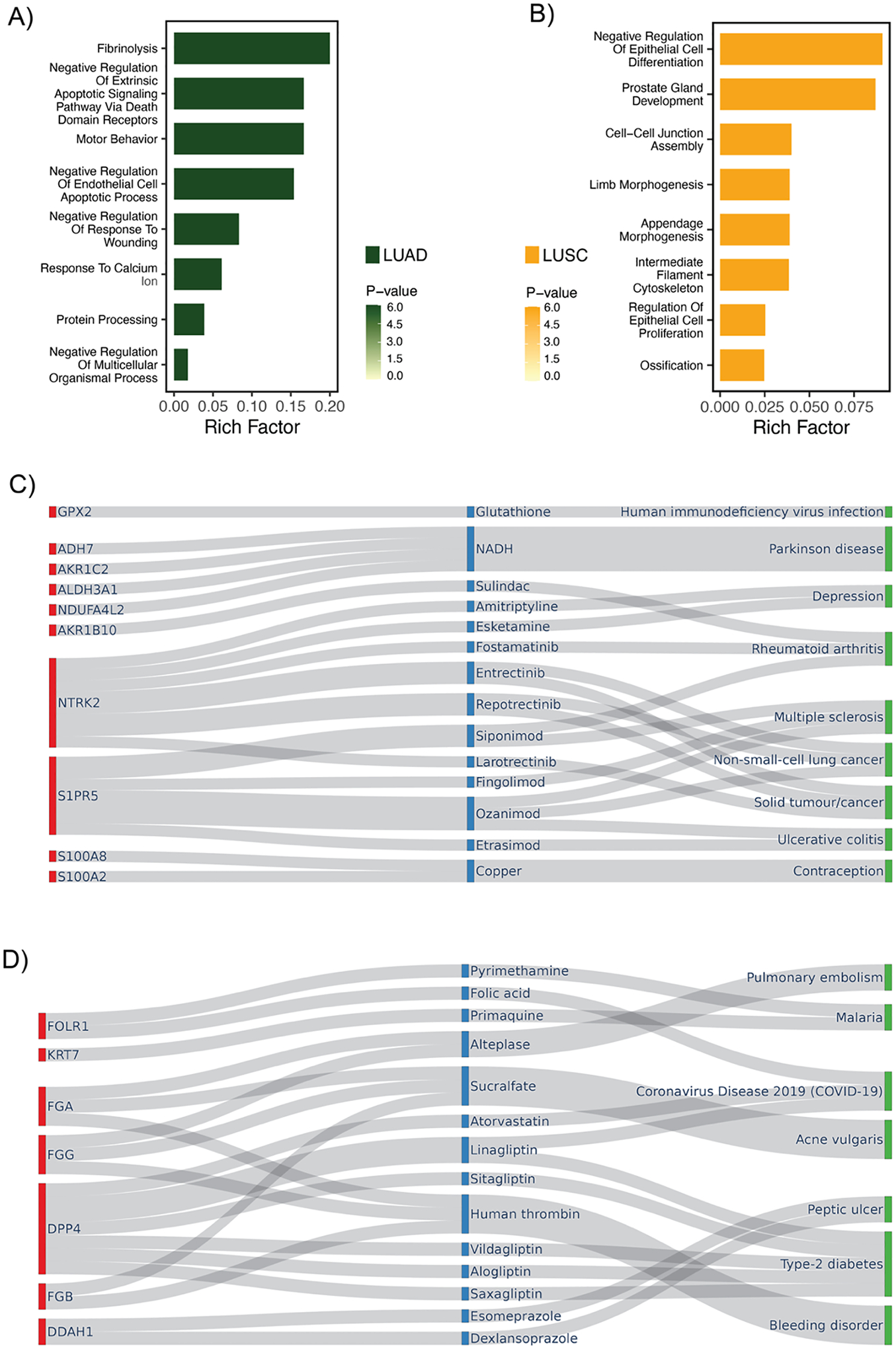
NSCLC subtype-specific marker genes characterization by pathway analysis and gene-drug-disease network studies. A,B) GO pathway enrichment analysis of marker genes for LUAD (A) and LUSC (B), as identified by RPSLearner, revealing subtype-specific biological processes. Rich Factor represented the percentage of genes in the specific GO terms that were identified as DEGs. C,D) Network diagrams illustrated potential drugs repurposing for LUAD (A) and LUSC (B), suggesting their possible application in cancer therapy. Red: Target gene, Blue: Drug, Green: Disease.

To elucidate the biological relevance of the biomarkers identified by RPSLearner prediction, we implemented gene ontology (GO) enrichment analysis using ClusterProfiler (V3.21),^[28]^ selecting the top 8 significantly enriched pathways for each cancer subtype (Figure 5A,B). These GO pathways highlighted subtype-specific cellular functions and may reflect underlying mechanisms of tumorigenesis. For instance, “Negative Regulation of Extrinsic Apoptotic Signaling Pathway via Death Domain Receptors”, which was enriched in LUAD, aligned well with reports that LUAD frequently attenuates death-receptor signaling (e.g., FAS/TNFRSF10B-FADD-CASP8) through upregulation of inhibitors in LUAD.^[29]^

Functionally, dampened extrinsic apoptosis may contribute to immune evasion and therapy resistance. In LUSC, “Negative Regulation of Epithelial Cell Differentiation” was among the most significantly enriched terms. This pathway is closely linked to the cell-of-origin hypothesis for this subtype, consistent with findings by Ogden et al.^[30]^ who reported that genes involved in squamous differentiation represent a frequently deregulated axis in LUSC. Mechanistically, alterations in NOTCH signaling and NFE2L2/KEAP1 axis may further reinforce impaired differentiation and oxidative-stress adaptation typical of LUSC. Overall, the enriched pathways observed for each subtype appear to align with evolutionary trajectories and cellular origins of the respective tumor types. Additionally, to further explore the therapeutic potential of these newly identified biomarkers, we performed gene-drug-disease network studies through cross-referencing target genes with drug and disease associations in the Durgbank^[31]^ database and Therapeutic Target Database (TTD)^[32]^ for drug repurposing opportunities (Figure 5C,D). Candidate drugs were selected based on shared target genes with those approved for NSCLC and other solid tumors. For example, Etrasimod,^[31]^ an approved therapy for ulcerative colitis, targets S1PR5, a gene also targeted by several NSCLC drugs. Such drugs, as illustrated in our figures, may serve as promising candidates for subtype-specific cancer therapy.

## 3. Discussion

In this study, we introduced RPSLearner, a novel approach that combines RP for dimensionality reduction and a stacking learning framework to aggregate predictions from diverse base models. RPSLearner utilized RP to reduce the dimensionality of transcriptomics data. Unlike other dimensionality reduction approaches that distorted intrinsic sample-to-sample relationships, RP applied in RPSLearner was able to capture meaningful and accurate latent features from the transcriptomics data, efficiently reduce dimensionality while preserving intrinsic distances among samples. This was crucial for maintaining the structural integrity of the data, which is essential for accurate subtype prediction.

RPSLearner applied feature fusion, which concatenated multiple independent RP reduced features for a richer representation learning. Compared to score ensemble strategies used in conventional RP-based algorithms that merely integrated the model scores from multiple RP reduced results, feature fusion could represent a richer aspect of hidden information since it integrated multiview latent features, and each reduced feature was independently generated from the original transcriptomics data. These results suggested that it could efficiently exploit variances from multiple RP matrices, achieving more representative latent features for lung cancer subtype prediction. These results demonstrated that exploiting multiscale feature space could enhance the effectiveness and generalization ability of the model.

Another unique advantage of RPSLeaner is the stacking framework. RPSLearner leverages a *meta*-learner, specifically a deep linear neural network, to learn the concatenated RP features combined with multiple RP reduced results and diverse conventional ML models. The stacking framework leveraged the complementary strengths of diverse base models as well as the insights from the *meta*-learner. The *meta*-learner could learn and utilize the knowledge from others, leading to improved performance across multiple metrics. Our results emphasized that the predictions of the *meta*-model are better than those from each individual base model. The use of a stacking learning framework improves the robustness and capacity of the prediction model through leveraging various base models with heterogeneous abilities to explore the same data.

RPSLearner demonstrated superior performance compared to state-of-the-art lung cancer subtype prediction methods. We tested the other methods, such as MCFS + SVM from Huang et al.^[12]^ which applied MCFS for feature selection, and SVM for cancer subtype prediction. Comparative analysis revealed that RPSLearner consistently showed higher performance across various metrics. A reasonable explanation for RPSLearner’s superior performance was that the application of concatenated RP features and stacking learning to leverage both RP transformed features and predictions from diverse base models, rather than simply averaging the prediction results.

RPSLearner exhibited biological interpretability to identify characteristic biomarkers from NSCLC subtypes. RPSLearner not only extracted meaningful hidden features from transcriptomics data but also offered insights about the detection of novel potential biomarkers. Using DEGs, we identified subtype-associated signals that are largely concordant with NSCLC biology, including EMT,^[33]^ abnormal cell-cell junction,^[34]^ and interferon signaling,^[35]^ frequently implicated in LUAD/LUSC. These pathway enrichment analyses provided a mechanistic context for predictions, holding potential for feasible validation through immunohistochemistry. These results highlighted its potential for clinical application. Besides, the method could be extended to more cancer types, as each cancer type may present distinct molecular and genomic characteristics, making it essential to evaluate the effectiveness in universal cancer subtypes prediction.^[36]^ Moreover, the method could also be extended to other tasks related to cancer diagnosis, such as cancer progression prediction, where the goal shifts from classifying subtypes to predicting tumor growth or patient prognosis over time.^[37]^ By incorporating longitudinal data, such as multiple time points of gene expression data, RPSLearner could provide valuable insights into disease progression, ultimately informing personalized treatment strategies.^[38]^

To interpret RPSLearner, we applied SHAP to the input of the model and ranked features by global importance (Figure S5, Supporting Information). The top 10 RP components together accounted for less than 20% of the cumulative attribution (mean SHAP value across samples), indicating that predictive variance was distributed across many components rather than dominated by a few. This contrasts with PCA, for which the variances are dominated by a few most significant principal components. By leveraging multiple RPs and a stacking model, we could accumulate discriminative and diverse information derived from multiple RPs to eventually improve prediction performance by RPSLearner.

Despite the innovations and contributions we mentioned in the previous sections, we also notice several potential limitations of this study. First, although the multiple times of statistical cross-validation tests in TCGA offer a robust estimate of model performance, independent validation by external datasets is always ideal to further demonstrate the superiority of RPSLearner. Independent validation on non-TCGA cohorts will quantify out-of-domain performance, reduce optimism, and demonstrate transportability. We will explore external datasets and implement our model on them for further validation in our future work. Second, the cohort is skewed toward white patients and elder age groups, which may constrain translation applicability to underrepresented groups (e.g., nonwhite populations and younger patients). Besides, while stacking multiple RP views improves NSCLC subtyping performance, the clinical interpretability might be compromised due to the complicated architecture of RPSLearner. To address this limitation, we have adopted the SHAP-based interpretability analysis to improve the clinical interpretability. However, we admit that even such a strategy would not make our complex stacking model fully interpretable. To this end, future work could be focused on exploring sparsity-promoting *meta*-learners or pathway-annotated RP to enhance parsimony without compromising accuracy.

## 4. Conclusion

In this article, to address the concerns in NSCLC subtype prediction, we developed RPSLearner, which combines RP and stacking learning for effective and accurate classification. It effectively reduced the dimensionality while preserving sample-to-sample distances through RP and integrated concatenated RP features and predictions from diverse models through stacking learning. RPSLearner succeeds in boosting classification prediction with higher accuracy, F1, and AUC metrics than conventional ML models and state-of-the-art methods. RPSLearner utilized a feature fusion strategy, which exhibited better performance than score ensemble approaches in subtype prediction. RPSLearner’s results are interpretable that the expression of DEGs aligns well with the published literature, which also offers insights about potential novel biomarkers. This framework could potentially be extended to subtype identification of other cancers.

## 5. Experimental Section

### Dataset:

Bulk RNA-seq data for LUAD and LUSC were retrieved from the NCI Genomic Data Commons (GDC) database^[39]^ with 9 different data sources (Figure S1, Supporting Information). After excluding samples with incomplete expression profiles and yielded a final dataset of 1,333 cases was yielded, with 731 LUAD and 602 LUSC cases, respectively. Cohort characteristics like race, age, and pathologic stages are summarized in Table S1–S3, Supporting Information. Approximately, two-thirds of patients were White (907/1,333; 68%). Among those with known age, three-quarters were over 60 years (756/1,001; 75.5%). Most tumors presented at an early stage (I-II: 1,034/1,333; 77.6%). The raw read counts of all RNA-seq samples were normalized to Transcripts Per Million (TPM). After that, the data were log-transformed without further quantile normalization and batch effect correction.

### RPSLearner:

Specifically, considering a data matrix **X** of size *n × d*, where *n* was the number of samples and *d* was the original dimensionality. RP found a random matrix ${R\in R}^{\text{d}\times \text{k}}$, with *k* ≪ *d*, such that the transformed data matrix ${Y=X\cdot R,\in R}^{\text{n}\times \text{k}}$, captured the hidden geometry of the original data **X** in the reduced dimension *k*.

One common choice of **R** is a Gaussian RP matrix.^[40]^ Each entry *r*_ij_ in **R** is independently sampled from a Gaussian distribution 𝒩(0, σ^2^), where σ^2^ is set to $\frac{1}{\sqrt{k}}$, ensuring that the resulting projected vectors are suitable to Johnson–Lindenstrauss Lemma. In other words,

$$r_{\text{ij}}\sim 𝒩\left(0,\frac{1}{k}\right)\;\text{for}\;i=1,2\ldots ,d;j=1,2,\ldots ,k$$

Then for a data point ${x\in R}^{d}$, its *k*-dimensional projection is ***y* = *x*** · **R**. With high probability, the Euclidean distances among samples in the projected space $R^{k}$ will be close to those in original space $R^{d}$.

Following RP-based dimensionality reduction, the transformed feature vectors were fed into diverse machine-learning base models, each generating unique predictive labels. These base models encompassed a range of algorithms, including KNN,^[41]^ RF,^[42]^ LightGBM,^[43]^ Extra Trees,^[44]^ Catboost,^[45]^ XGBoost,^[46]^ Neural Networks,^[47]^ thereby capturing a wide spectrum of data patterns and interactions. Then, RPSLearner aggregated the predictions from these base models with transformed feature vectors using a stacking *meta*-learner, specifically a MLP, which synthesized the individual predictions and features to enhance overall classification performance. This stacking strategy not only mitigated the biases inherent in individual models but also leveraged their complementary strengths, resulting in a more robust and accurate subtype prediction.

In our stacking framework, we employed a diverse set of base classifiers, each generating its own prediction. Let ${x\in R}^{d}$ denote an input feature vector from the dataset and let the set of possible class labels be *ℓ* = {1, 2, …, *C*}. Each base classifier *f*_*i*_(·) returns its predicted label ${\hat{p}}_{\text{n}}\in \ell$

The stacking method required a *meta*-learner model to integrate the predictions from various base models. Let there be *B* base models {*f*_1_, *f*_2_, …, *f*_N_}. Each model *f*_N_ could output a predicted label. ${\hat{p}}_{\text{n}}$ For numerical stability and richer information, we concatenated all base model predictions, appending the original input ***x*** to form a composite feature $z\left(x\right)=\left[x,{\hat{p}}_{1},\ldots ,{\hat{p}}_{\text{N}}\right]$. The final prediction $\hat{y}$ could be formulated as

$$\hat{y}=\underset{c\in C}{\text{argmax}}\;g\left(z\left(x\right)\right)$$

where *g*(*z*(***x***)) represented the stacking *meta*-learning model and argmax function aim to get the prediction label with the highest probability.

A 4-layer MLP was used as the *meta*-learner in RPSLearner with the numbers of neurons in the hidden layers as 128-64-32-16, ReLU activation function, and 0.2 dropout rate. For the training setting of the *meta*-learner, we applied an Adam optimizer (learning rate = 1e–4) and weighted random sampling^[48]^ for batch loading.

For robust and unbiased evaluation of RPSLearner, we used 10 times stratified fivefold cross-validation tests.

### DEG Analysis and GO Pathways for RNA-seq Data:

After obtaining the subtype prediction from RPSLearner, we grouped the samples according to the predicted labels. We used the lmFit and eBayes functions in the R *limma* (V3.64.0) package to identify the significantly differentially expressed genes defined by *P* value < 0.05 and log_2_ Fold Change > 1. Next, based on these differentially expressed genes, we used a Venn diagram to show their common and unique DEGs for each NSCLC subtype. Then, we implemented GO analysis through ClusterProfiler^[28]^ (V3.21) based on the pathway database attached to it. We exploited the *enrichGO* function with the *p*-value threshold of *P* < 0.05 to obtain the GO annotation of the DEGs and selected the top 8 items for each subtype to present.

### Target-Drug-Disease Linkage:

The drug and disease linkages were extracted from the DrugBank database.^[31]^ And the target-disease linkages were curated in the Therapeutic Target Database^[32]^ (TTD). Cross-referencing the identified genes against these pharmacological datasets yielded a refined list of drugs for potential repurposing as novel therapeutic strategies against each cancer subtype.

### RP Component Interpretability Analysis:

We implemented the SHAP^[49]^ (v0.48) package for RP components interpretability analysis. Due to the RPSLearner is a *meta*-learning method, we applied the *KernelExplainer* function to get the subtype-specific contributions of ensembled RP components. Here, we conducted a 20% hold-out training strategy to train RPSLearner with 400 RP dimensions and one RP matrix. After that, the top 10 RP components were extracted to further observe their contributions to each sample.

## Supplementary Material

Supplement Figs and tables

Supporting Information is available from the Wiley Online Library or from the author.

## Data Availability

Research data are not shared
