# Supplementary material for: RPSLearner: A Novel Approach Based on Random Projection and Deep Stacking Learning for Categorizing Non-Small Cell Lung Cancer
Source: Adv Intell Syst. Author manuscript; Available in PMC 2025 Dec 4. (PMC12674606; doi:10.1002/aisy.202500635)
Supplement: Supplement Figs and tables [file NIHMS2121493-supplement-Supplement_Figs_and_tables.pdf]

Supporting information

Supplement figures

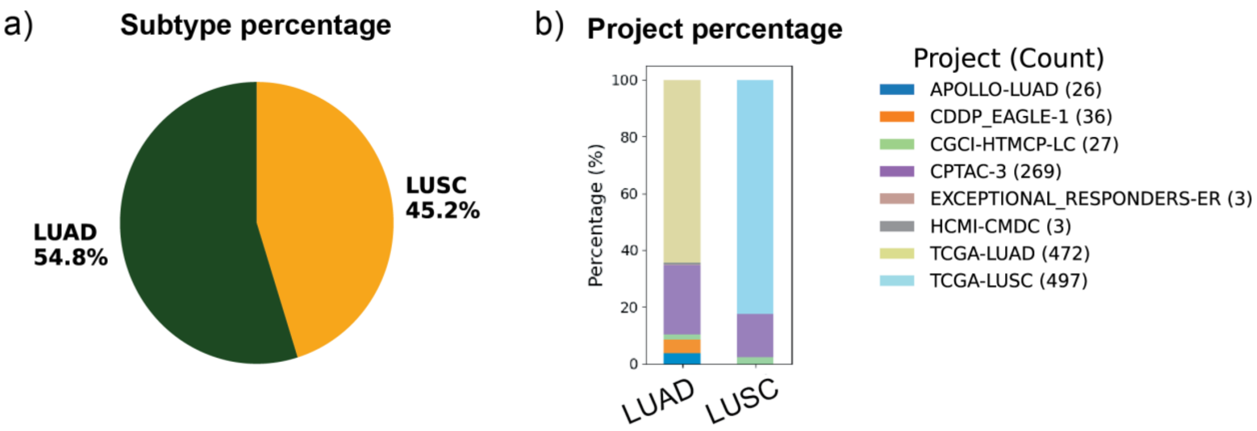

Supplement Figure S1. Overview of the NSCLC RNA-seq cohort from TCGA database. a Distribution of lung cancer subtypes. LUAD: Lung adenocarcinoma, LUSC: Lung squamous cell carcinoma. **b)** Breakdown of sample counts by TCGA project within each subtype.

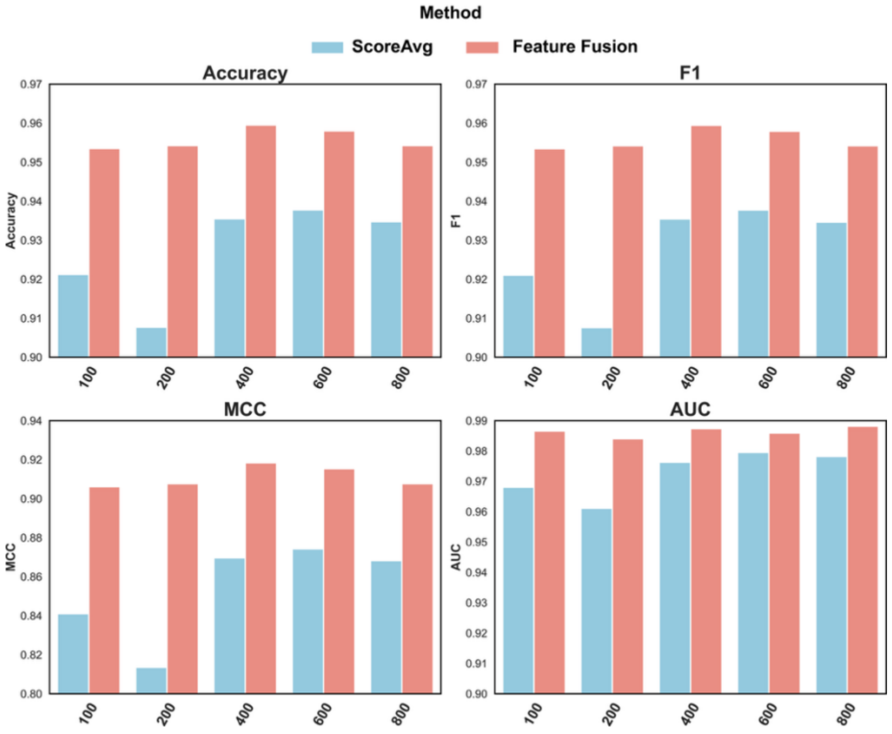

Supplement Figure S2. Score averaging vs. feature fusion across projection dimensions. Performance comparison of probability score averaging (ScoreAvg) and feature fusion (concatenated RP features trained with a single classifier) across RP

dimensions under an a 20% hold-out training split. MCC: Matthews Correlation Coefficient. AUC: Area under ROC curve.

a)

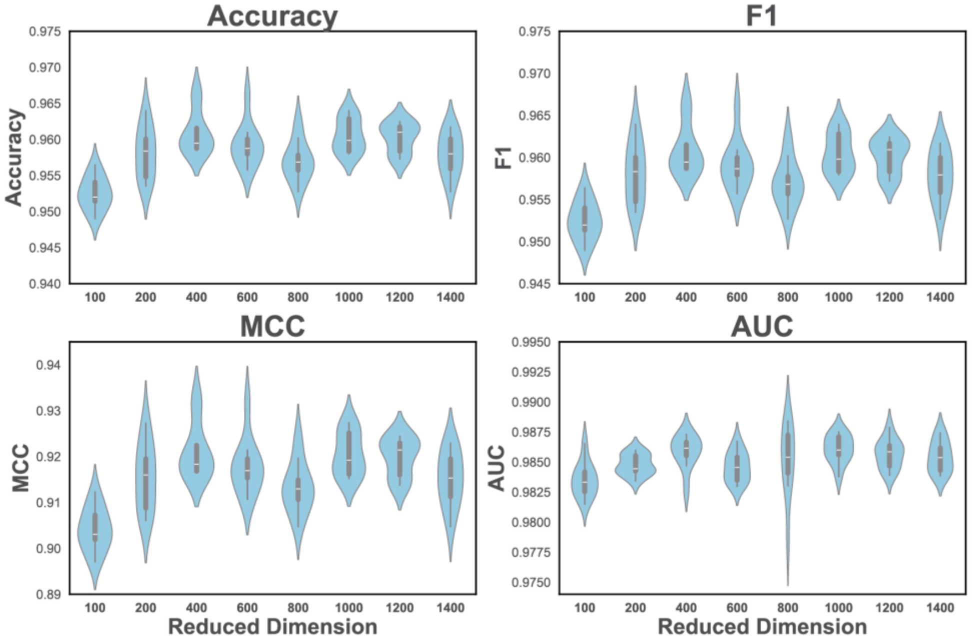

b)

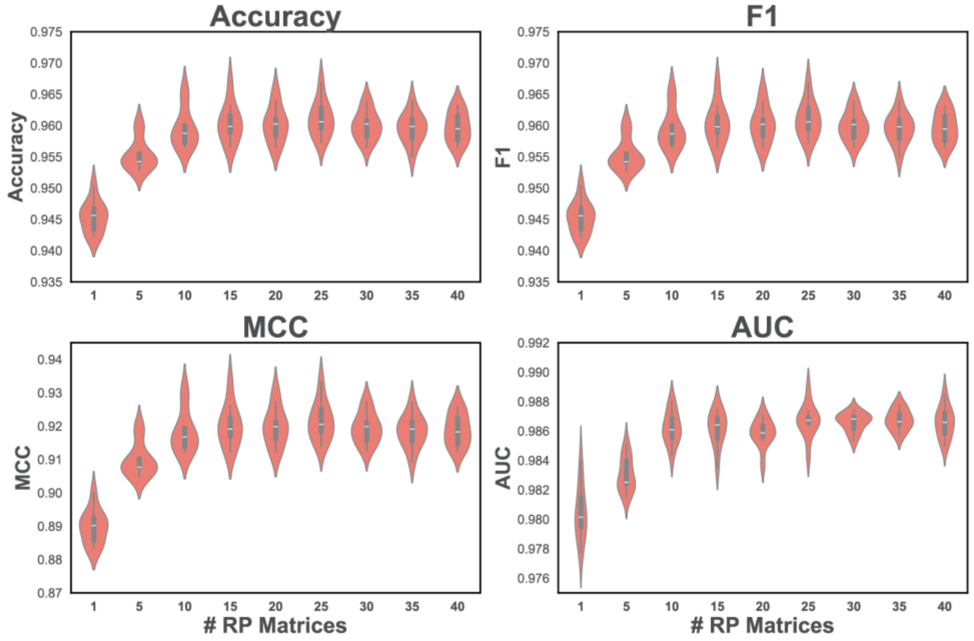

Supplement Figure S3. RP component setting tuning of RPSLearner. a) Model performance across varying RP dimensions. **b)** Model performance as a function of the number of RP matrices. Metrics abbreviations as above.

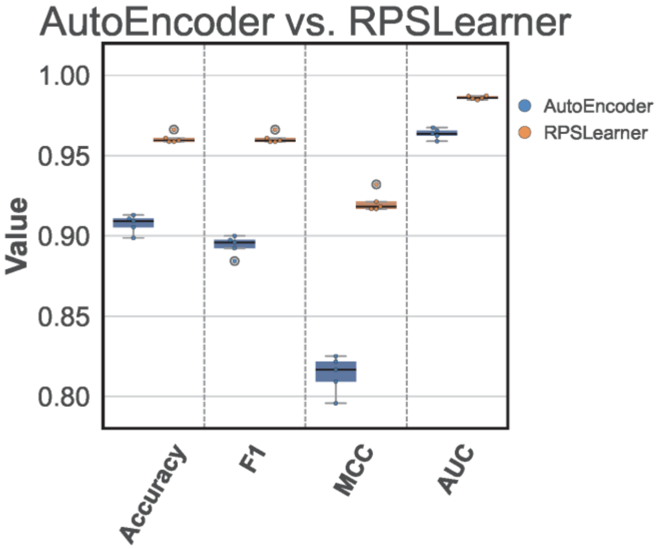

Supplement Figure S4. Deep-learning baseline vs. RPSLearner. Comparison of an auto-encoder (AE) baseline model against RPSLearner across accuracy, F1, MCC, and AUC under a 5-fold CV split. Metrics abbreviations as above.

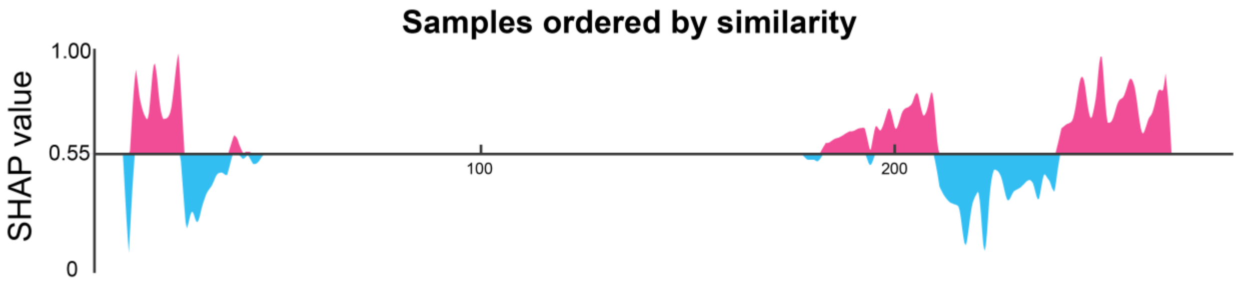

Supplement Figure S5. SHAP interpretability of top 10 RP components. Subtype-specific SHAP values summarizes showing per-sample contributions of the ten most influential RP components in a 20% hold-out split testing.

Supplement tables

Table S1 Race composition by NSCLC subtype (column % within subtype)

Race group	LUAD n (%)	LUSC n (%)	Total
White	491 (36.8%)	416 (31.2%)	907
Non-white (known)	178 (13.4%)	74 (5.5%)	252
Unknown/Not reported	62 (4.7%)	112 (8.4%)	174
**Total**	**731**	**602**	**1333**

Table S2 Binned age distribution by NSCLC subtypes (column % within subtype)

Age range	LUAD n (%)	LUSC n (%)	Total
<40	4 (0.3%)	1 (0.1%)	5
40–60	144 (10.8%)	96 (7.2%)	240
60–80	328 (24.6%)	375 (28.1%)	703
80+	26 (2.0%)	27 (2.0%)	53
Unknown	229 (17.2%)	103 (7.7%)	332
**Total**	**731**	**602**	**1333**

Table S3 AJCC pathologic stage (I-IV, Unknown) by NSCLC subtypes (column % within subtype)

Stage	LUAD n (%)	LUSC n (%)	Total
I	380 (28.5%)	280 (21.0%)	660
II	172 (12.9%)	202 (15.1%)	374
III	133 (10.0%)	101 (7.6%)	234
IV	29 (2.2%)	8 (0.6%)	37
Unknown	17 (1.3%)	11 (0.8%)	28
**Total**	**731**	**602**	**1333**
